# Current News and Opinion

**Published:** 1887-07

**Authors:** 


### Current News and Opinion.

### AMERICAN DENTAL ASSOCIATION.

The twenty-seventh annual meeting will be held at Niagara Falls, commencing Tuesday, August second, 1887. The officers having decided to change the place of meeting from Asheville, North Carolina, to Niagara Falls, it is hoped that we may have a large attendance. The principal reasons for making the change were, that many dentists residing in the South desired to attend the meeting of the Southern Dental Association, and also the International Medical Congress, to be held in Washington, D. C., September 5, 1887, both gatherings coming close together. The American Dental Association would, they thought, have a larger meeting in the North this year than would be the case had they gone to Asheville. Next year the Association may meet in the South, when it is believed we will have many accessions to our ranks You are especially urged to be present at Niagara, and help to make this one of the best meetings ever held by the Association. Arrangements are being made with railroads and hotels to secure their best rates, due notice of which will appear in the July issues of the journals.

W. W. ALLPORT, President.
GEO. H. CUSHING, Recording Sec'y.
A. W. HARLAN, Corresponding Sec'y.

All railroads west of Chicago, and from Chicago leading to Niagara Falls, will carry members and delegates of the American Dental Association at one and one-third fares.

#### READ THE RULES CAREFULLY.

"Each delegate must purchase a first-class ticket to the place of meeting, for which he will pay the regular fare, and upon request the ticket agent will issue to him a certificate of such purchase.

"If through tickets cannot be procured at the starting point, delegates will purchase to the most convenient point where such through tickets can be obtained, and re-purchase through to place of meeting, requesting a certificate from the ticket agent at the point where the re-purchase is made.

"Tickets for the return journey will be sold by the ticket agents at the place of meeting at one-third the highest limited fare, only to those holding certificates signed by the ticket agent at point where through ticket to place of meeting was purchased, and countersigned by the secretary or clerk of the convention certifying that the holder has been in attendance upon the convention."

Tickets are good—going, three days before the meeting; and returning, three days after its termination.

Equally favorable rates are secured from the south and east

A. W. HARLAN,
Chairman Executive Committee.

The annual meeting of the National Association of Dental Examiners will be held at Niagara Falls, on Monday, August 1, 1887, at 3 P. M.

FRED A. LEVY, Sec'y.

---

### FIRST DISTRICT DENTAL SOCIETY, STATE OF NEW YORK.

### COPY OF RESOLUTIONS ADOPTED AT A REGULAR MEETING HELD IN NEW YORK CITY, ON TUESDAY EVENING, JUNE 7, 1887.

WHEREAS, Members of this society are threatened with suits for damages and injunctions if certain Letters Patent for alleged improvements in dentistry are not recognized, the validity of which has been gravely questioned, and the right to use is wholly refused, or terms and conditions imposed which would be a heavy tax upon the profession and the community for many years; and—

WHEREAS, It would be unjust for one or two members to bear the labor and heavy expense attendant upon determining how far the pretensions of such patentees ought to be respected, therefore—

*Resolved*, That each member of this society be requested to contribute five dollars to its treasurer towards a protective litigation fund, to be expended as the officers of the society, or a special committee, direct.

*Resolved*, That a copy of these resolutions be sent to other dental societies with a suggestion of the imperative necessity of financial co-operation, if effective resistance is to be made to the preposterous demands now made upon dentists by patentees.

The following resolution was also adopted at the same meeting:

*Resolved*, That this society recognizes the value of the proposed meeting of the Dental and Oral Section of the International Medical Congress, and hereby expresses its approval of the same and guarantees moral support to the movement That we recommend that its members lend such assistance as may be needed by the officers of the Section, thereby promoting the best interests of the profession.

B. C. NASH, Secretary. W. W. WALKER, President.

The resolutions were also adopted by the AMERICAN ACADEMY OF DENTAL SURGERY, of New Jersey, at a special meeting held on the evening of June 2, 1887. W. W. WALKER, President.

These resolutions were also unanimously adopted at the annual meeting of the Buffalo City Dental Association, held June 25th, and an assessment of five dollars for each member was agreed upon. Dr. A. P. Southwick was appointed custodian of the fund.

HERBERT A. BIRDSALL, D. D. S., Sec'y.

### SECOND DISTRICT DENTAL SOCIETY.

COPY OF RESOLUTIONS ADOPTED AT A REGULAR MEETING HELD IN THE CITY OF NEWBURGH, ON MONDAY, JUNE 13, 1887.

WHEREAS, Members of this society are threatened with suits for damages and injunctions if certain Letters Patent for alleged improvements in dentistry are not recognized, the validity of which has been gravely questioned, and the right to use is wholly refused, or terms and conditions imposed which would be a heavy tax upon the profession and the community for many years; and—

WHEREAS, It would be unjust for one or two members to bear the labor and heavy expense attendant upon determining how far the pretensions of such patentees ought to be respected, therefore—

*Resolved*, That the sum of three hundred and fifty dollars (that being $5 per member) be contributed from the treasury of this society towards a protective litigation fund, to be expended as the following committee of three shall direct: Dr. O. E. Hill, of 160 Clinton Street, Brooklyn; Dr. A. H. Brockway, of 13 Greene Avenue, Brooklyn, and Dr. J. P. Geran, of 171 Greene Avenue, Brooklyn.

The following resolution was also adopted at the same meeting:

*Resolved*, That this society recognizes the value of the proposed meeting of the Dental and Oral Section of the International Medical Congress, and hereby expresses its approval of the same and guarantees moral support to the movement. That we recommend that its members lend such assistance as may be needed by the officers of the Section, thereby promoting the best interests of the profession. F. T. VAN WOERT,

Rec. Secretary.

### THE AMERICAN MEDICAL ASSOCIATION.

*Editor Independent Practitioner:*

Enclosed please find copy of resolutions introduced by Dr. N. S. Davis, Chicago, (Pres. of International Medical Congress) and adopted by the American Medical Association, June 10, 1887. With this action the graduates of reputable dental colleges in the United States will bear the same relation to medicine and the American Medical Association, as the English surgeons do to medicine and the British Medical Association By the strenuous efforts of men like Dr. Allport, this question of the recognition of the D. D. S. by the M. D. has been finally settled. This action will unquestionably prove to be a benefit to both physician and dentist. Yours very truly,

E. S. Talbot,
Sec'y Section of Dental and Oral Surgery.

*Resolved,* That the regular graduates of such dental and oral schools and colleges as require of their students a standard of preliminary or general education and a term of professional study equal to the best class of the medical colleges of this country, and embrace in their curriculum all the fundamental branches of medicine, differing chiefly by substituting practical and clinical instruction in dental and oral medicine and surgery, in place of practical and clinical instruction in general medicine and surgery, be recognized as members of the regular profession of medicine, and eligible to membership in this Association on the same conditions and subject to the same regulations as other members.

Dr. Davis in introducing the resolution said he wished to explain its object. There are two objects to be had in view: first to relieve a degree of embarrassment that exists between the regular profession as we consider it, and the profession of dentistry. The department of dental and oral surgery is a part of the profession of medicine as much as the department of ophthalmology or otology or any other ology. Our teeth and mouth are a part of our system as much as any other part, and are used more than any other. The embarrassment is this; that in the history of the development of dentistry it originated mostly in mechanical operations. Steadily it has advanced, and in years gone by—quite a number of years ago—our lamented S. D. Gross made a proposition that an oral and dental section be provided as a section in this Association. It was seconded by Dr. Sayre and myself, and it was organized. The International Medical Congress of 1881 provided a section for dental and oral surgery. The Congress to be held in Washington has done the same thing, and it will be one of the most thorough and best organized sections in the Congress. There is an embarrassment in this respect. It is to know just who and by what line of demarkation those engaged in that department shall be recognized as members of the regular profession Now it is proposed to make a line and draw it where this resolution says; that all those who are qualified by general education and a course of study equal to the best medical colleges, a curriculum embracing the entire fundamental principles of medicine, with the provision that instead of special instruction in clinical medicine and surgery, instruction may be had in dental and oral surgery, shall be recognized as members of the profession of medicine. It will take away a sort of embarrassment. There is a more far-reaching and more valuable underlying object in this resolution, and that is that to be recognized as a member of the profession, if this resolution is adopted by this body they must have the education received in schools that require these qualifications, and it will make a strong lever to lift up the course of study in the dental schools. Such are my reasons for bringing up the resolution. I will say nothing more on the subject.

The motion was made that the resolution be adopted by the Association, and it was carried unanimously.

### INTERNATIONAL MEDICAL CONGRESS.

### EXPLANATORY STATEMENT.

Learning that there is still uncertainty in the minds of some as to the rights, privileges and status in the Ninth International Medical Congress of the regular graduates of the class of dental colleges specified in the resolution passed at the late meeting of the American Medical Association, it is deemed right and proper to say that the graduates of all dental colleges whose requirements conform to that resolution are considered as members of the medical profession, and are eligible to membership in the American Medical Association. They may register and take out a card of membership according to general regulations, precisely upon the same terms and under the same conditions as those who hold the degree of M. D.

Dental practitioners who, according to this resolution, are not graduates but are recommended by members of the council, become members of the Congress upon invitation, authorized by the Executive Committee.

The members of the seventeenth Section differ in no respect from those of any other Section of the Congress, and have the same rights, privileges and status.

Without doubt a large proportion of those who have received notice that they will be invited are fully qualified according to the above resolution. Whether all are, cannot now be determined, nor is it important that it should be. It is desirable to have present all who have been selected for membership. It is also hoped that those who are qualified according to the resolution will become members, and those who have not this special qualification but have high attainments in the science and practice of dentistry, may become members as any other scientific men.

Invitations will soon be sent to all who have signified a willingness to accept an invitation, and though for many this is not necessary, yet it is thought best to adhere to the original plan.

Should there be any who have delayed in complying with the request of the circular from Dr. F. H. Rehwinkel in regard to invitations, they are requested to do so speedily. J. TAFT, Chairman of Section 17, I. M. C.

---

### WHAT IS THE CAUSE ?

I have repeatedly asked the question, Why is it that certain symptoms appear in connection with the wearing of *red* rubber plates, which I never find when other colored rubbers or metal plates are worn, and which disappear upon the removal and substitution of other materials ?

I refer to the serious stomach troubles and chronic diarrhœa, lasting as long as the plates are worn, and disappearing as if by magic when they are permanently removed.

There are also many cases where the corners of the mouth are constantly sore from the same cause Within two weeks I have had a case of this kind. The lady had worn a red rubber plate four years. I noticed the corners of the mouth were sore, and asked how long they had been so. She replied, "Ever since I wore the plate." I made her a gold plate, and upon calling a week later the trouble had disappeared.

I mention this case because it is recent, but I have known many others.

I ask again if some one will explain the cause, bearing in mind the fact that I have never seen these symptoms in connection with anything but the *red* rubber.

L. P. HASKELL.

---

### AMERICAN DENTAL SOCIETY OF EUROPE.

The annual meeting of The American Dental Society of Europe will be held at the Hotel zum Riesen, Coblence, on Thursday, Sept. 1, 1887, at 10 A. M , and continue for two or three days.

*President*—E. P. George.

*Vice-President*—C T Terry.

*Secretary*—E. A. Galbreath.

*Treasurer*—W. Sachs.

*Microscopist*—W. D. Miller.

*Membership Committee*—C. T. Terry, F. Foester, L. C. Bryan.

*Executive Committee*—E. P. George, B. Cohen, N. S. Jenkins.

The work has been divided this year into sections, which will report in the following order, viz:

I. Operative Dentistry—Charles Kingsley, Chairman.

II. Dental Materia Medica—Wm. St. George Elliott, Chairman.

III. Prosthetic Dentistry—W. M. Patton, Chairman.

IV. Dental Education—Geo Cunningham, Chairman,

V. Dental Pathology and Histology—W. D. Miller, Chairman.

Clinics illustrating various methods of operating may be expected.

There will be an exhibition of instruments and of models and appliances for plate-work and regulating cases.

Gentlemen who design contributing papers and who have not already sent in the titles of their essays are requested to communicate them to

E. P. GEORGE,
5 Marien Str.,
Frankfort a|M.

---

### NEW JERSEY STATE DENTAL SOCIETY.

The seventeenth annual session of the New Jersey State Dental Society will be held at Asbury Park, N. J., "Coleman House," July 20, 21, 22. It is expected to be the largest meeting ever held by the society. Many interesting papers by eminent dentists in the profession throughout the country will be read. The clinics will be made the most interesting adjuncts of the sessions, about twenty of the most skilled in the profession having signified their intention to attend and give clinics.

The board will be $2.50 and $3.00 per day, the accommodations being of the best. The Secretary will mail programmes to those desiring, every member of the profession being cordially invited to attend.

CHARLES A. MEEKER, D. D. S , Secretary.

### SIXTH DISTRICT DENTAL SOCIETY.

At the regular annual meeting of the Sixth District Dental Society of the State of New York, in the city of Binghamton, Tuesday May 10th, 1887, the following officers were elected for the ensuing year:

*President*—E. D. Downs, Owego.

*Vice-President*—M. H. Fish, New Berlin.

*Secretary*—Myron D. Jewell, Richfield Springs.

*Treasurer*—F. B. Darby, Elmira.

*Censor*—A. M. Holmes, Morrisville.

*Delegates to State Society for Four Years*—M. H. Fish, New Berlin; G. H. Smith, Cortland.

MYRON D. JEWELL, Secretary.

---

### WISCONSIN STATE DENTAL SOCIETY.

The Wisconsin State Dental Society will hold its seventeenth annual meeting at Milwaukee, Wis., beginning Tuesday, July 19, 1887, continuing for three days.

The State Board of Dental Examiners will meet daily during the sessions to examine and register applicants.

Dental and medical practitioners are cordially invited to attend.

W. S. SULLIVAN, D. D. S., Secretary.

23 E. Main St., Madison, Wis.

---

### SUSQUEHANNA DENTAL ASSOCIATION.

The officers elected at the last annual meeting were as follows:

*President*—J. D. Wingate, Carbondale.

*Vice-President*—C. S. Beck, Wilkes Barre.

*Corresponding Secretary*—C. T. Meaker, Carbondale.

*Recording Secretary*—G. W. Klump, Williamsport.

*Treasurer*—Henry Gerhart, Lewisburg

---

### NATIONAL ASSOCIATION OF DENTAL FACULTIES.

The National Association of Dental Faculties will meet at the Ebbitt House, in Washington, D. C., on Saturday, September 3, 1887, at 10 o'clock, A. M.

By order of the Executive Committee. C. N. PEIRCE, President.

H. A. SMITH, Secretary.

---

### MARRIED.

At the residence of the bride's father, No. 54 Stimson Place, Detroit, Mich., on the evening of June 15, 1887, Miss Jessie, only daughter of Dr. Geo. L. Field, to John R. Campbell, of Detroit.

The most earnest and heart-felt wishes of a wide circle of friends go with the charming and amiable bride and the accomplished groom, and if the prayers of all who know them are answered, the bark of the happily wedded pair will sail in none but placid seas, and be wafted by only prospering gales

### USELESSNESS OF LIME PHOSPHATE AS A BONE AND TISSUE FORMER.

M. Logeais quotes the experiments of Lehman, Heiden and Veiske, who claim to have demonstrated that the phosphates of lime added to the food are not absorbed, but are excreted in their entirety in the dejections. Dujardin Beaumetz is quoted as saying that this salt has no action whatever, while neither the phosphate of lime nor phosphoric acid is absorbed. To this may be added the statement of Nothnagel and Rossbach, " Never, as far as we know, has a case of rickets been cured by the administration of lime." "Everywhere there is a lack of trustworthy and extensive observations," as to its utility. All the forms of lime-salts used in practice are, according to M. Logeais, precipitated and made insoluble as soon as they pass into the stomach. They are, therefore, taken up, if at all, in the stomach alone, and the amount absorbed here is small. The practical conclusion that lime-salts of all kinds are therapeutically useless, is one which deserves the attention of the many physicians who are constantly prescribing them in various conditions. Very many dentists make it a routine practice to advise their patients to take lime-salts, especially in pregnancy; it is important that they should note that such a measure is probably absolutely without use.—*British Journal Dental Science*,

---

### MELTING POINT OF METALS—FAHRENHEIT.

| | | | | | |
|---|---|---|---|---|---|
| Aluminum | 1292 | Gold | 2485 | Mercury | 40 |
| Antimony | 797 | Indium | 348 | Nickel | 2912 |
| Arsenic | 365 | Iron, wrought | 2786 | Potassium | 143 |
| Bismuth | 507 | Iron, cast | 2192 | Platinum | 4712 |
| Cadmium | 608 | Iron, steel | 2552 | Silver | 1904 |
| Cobalt | 2192 | Lead | 617 | Tin | 455 |
| Copper | 1995 | Magnesium | 455 | Zinc | 773 |

---

SEABURY & JOHNSON, (Seabury Pharmacal Laboratories) New York, that wide awake and reliable firm, have added to their list of laboratory products a full line of Medicated and Antiseptic Soaps of a high degree of excellence. Conspicuous in this addition are their one and five per cent. Hydronaphthol Soaps; the former for the Toilet, the latter for medicinal and antiseptic purposes. These Soaps are prepared from the purest ingredients obtainable, highly scented, entirely neutral, containing no free alkali, acid or impure fat. Dr. C. W. Allen, of N Y., and other prominent skin specialists recommend these Hydronaphthol Soaps very highly in the treatment of ulcers, eczema scabies, impetigo, pruritus, pityriasis capitis, alopecia, favus, etc., etc. Aside from their healing powers these Soaps also impart their well-known powerful antiseptic and disinfectant properties to the waters in which they are used, and in this way free havens, bath-tubs, cesspools, sinks, etc., etc., from all unpleasant odors and foul gases.

The United States now has a population of sixty millions,—more English-speaking people than exist in all the world beside; more than the whole population of the United Kingdom of Great Britain, plus twice the population of the British colonies. In 1770 the population of the United States was three millions.

In 1850 the wealth of the United States was £1,686,000,000; that of Great Britain £4,500,000,000. In 1880 the wealth of Great Britain had almost exactly doubled, and was £9,000,000,000, while that of the United States had reached £9,790,000,000.

In 1850 the United States had scarcely any manufactories as compared with Great Britain. In 1880 the latter amounted to £818,000,000, while those of America were $1,112,000,000,—nearly half that of the manufacturing wealth of all Europe.

---

Dr. J. N. Farrar, of New York, has for some years been engaged on a book, the first volume of which, it is rumored, will be published this year. It will be exclusively devoted to irregularities and abnormalities in teeth, and methods for their regulation. Dr. Farrar has long been considered an authority upon this subject, and his work will be looked for with interest. It will be published in two volumes of about 500 pages each. Its scope will be broad, exhaustive and liberal, and it will present an account of all the different plans for regulation that have been used, all fully illustrated by engravings. It will not be a mere compilation, but will be an entirely original work, and it is hoped that it will serve as a text-book and book of reference for both student and practitioner.

---

Dr. Fuerth, at the Polyclinic, in Vienna, vaccinated a number of children from the arm of a child who was at the time apparently healthy. Several days later the latter broke out with a syphilitic eruption. The children vaccinated from it had already been distributed in the country, but Dr. Fuerth feeling the great responsibility resting upon him, took special pains to hunt up each child and keep it under observation for some time. Not one of the children vaccinated from the syphilitic child had syphilis or showed any signs of it during the several months that they were watched.

Therefore, vaccination with pure lymph from a syphilitic child will not produce syphilis; and it is only when blood has been mixed with the lymph that syphilis is imparted in vaccination.—*Archives of Pediatrics.*

---

The Montreal Meeting of the Conn. Valley Dental Society, to be held July 19–23, should not be forgotten. It will be a memorable affair, not alone for the scientific and professional attractions that will be presented, but on account of the social features, to which particular attention will be given. Montreal is a hospitable place, and Montreal dentists propose to sustain the good reputation of their beautiful city. There are more objects of interest to visit, and finer exhibitions of natural scenery to be seen than we know of in almost any other locality. Visits to the Lachine rapids, and to quaint old Quebec and the wonderful Saguenay, are arranged for, to say nothing of the strictly local attractions. Secretary Geo. A. Maxfield, Holyoke, Mass., will give any desired information.

The American Dental Manufacturing Co , as may be learned by reference to their advertisement, have removed their office and salesroom to Nos. 1298 and 1300 Broadway, New York City. By unusual care and attention in both their manufactories and salesroom, this company have gained an enviable reputation, and their business has so increased that better facilities and more room were imperatively demanded. In their new quarters they will have increased opportunities for serving their patrons, and their past reputation is a sufficient guarantee that no pains will be spared or expense considered when thereby their specialties can be improved.

---

Dr T. W. Evans, of Paris, has, it is said, announced that he should, by his last will, devise sufficient funds for the proper endowment of a dental college. If this be so, how much better would it be if he would during his lifetime see the money properly bestowed, and himself attend to the organization of such a foundation. He would thus not only have the gratification of seeing the good work commenced and probably completed, but there would be a certainty that the money would be expended properly, and not given to greedy lawyers.

"The evil that men do lives after them;
The good is oft interred with their bones."

---

In Scribner's Magazine for July, Professor D. A. Sargent, M. D., of Harvard College, who is perhaps the best authority in this country on the general subject of Athletics, will publish, as the fruit of many years' practical experience, his first extended article in that field, under the title "The Physical Proportions of the Typical Man." In it Professor Sargent will give a standard of physical measurement, based on the measurements of ten thousand individuals. This furnishes a basis of comparison by which any person can gauge his proportions with those of the typical man. The article will contain charts for this purpose, founded on these observations.

---

Owing to the Teutonic Taste for raw ham, every German town has an official microscopist whose duty it is to examine all the hams placed in the market. At Schebitz, recently, several persons died of trichinosis, and the court, having found a verdict against the microscopist for careless performance of his duty, condemned him to two years' imprisonment.

It was certainly carelessness, for no microscopist can fail to recognize *trichina spiralis* if he makes a thorough examination of the meat.—*National Druggist.*

---

The following named gentlemen have been appointed officers in the Section of Dental and Oral Surgery of the International Medical Congress for Great Britain: J Smith Turner, Sec. T. H. Harding, C. Spence Bate, England; Wm. B. Macleod, Scotland; R. T. Stack, Ireland, Vice-Presidents

---

The May number of *The Transactions of the Odontological Society of Great Britain* contains a very elaborate and exhaustive paper upon "The Physiological Action of Cocaine on the Lower Animals and Man, and its use in Dental Surgery," by George Cunningham, B. A., D. M. D., L. D. S.

THE ATTENDANTS upon some dental meetings are regaled with "symposiums," whether to be served cold or hot the circulars do not state. A ' symposium" is probably intended only for the *elite*—for those who practice only "surgical" or "prosthetic" dentistry. "Symposium" is the latest fad, and according to Webster means a drinking party, it being derived from two Greek words which signify to drink together. We are decidedly in favor of symposiums, and like ours with plenty of sugar and water, but with no nutmeg.

DR. J. H. WOOLLEY, of Chicago, has devised a root canal dryer which is very effective. It consists of a copper broach, to which is attached a bulb that may be heated, and which in turn keeps the broach hot. This latter is introduced into the canal and allowed to remain there until the cavity is entirely dry. The perfect filling is then a comparatively easy matter. No root that is not entirely devoid of moisture can be perfectly filled, and a trial of Dr. Woolley's dryer will demonstrate its advantages.

DR. FRANK ABBOTT, chairman of the committee on Microscopy of the Section of Dental and Oral Surgery in the International Medical Congress, desires to hear from any one who is desirous of exhibiting specimens, reading papers, or in any way assisting in the microscopical department of the Section at the coming Congress. Address him at 22 West 40th Street, New York City.

PROF. DR. W. D. MILLER, of Berlin, has been elected a Vice-President of the International Medical Congress, and is therefore one of the general officers of the meeting. There is no one who is better entitled to the honor than is Dr. Miller. He will probably be present and will present a paper.

THE BOARD OF DENTAL EXAMINERS for Oregon, as finally arranged by the Governor, is as follows: Drs. J. R. Cardwell and John Welch for four years, and Drs. N R. Cox and S. J. Barber for two years. All are residents of the city of Portland.

AKRON RUBBER.—We wonder if dentists who are not already using this excellent material have availed themselves of the opportunity offered by the manufacturers for testing its merits? Samples are gratuitously sent wherever requested.

DR. A. M. DUDLEY, of Salem, Mass , commander of Salem Post of the G. A. R., has been making a visit to the southern battle fields of the rebellion, in charge of a large number of the citizens of Salem and Boston.

DR. G. V. BLACK, of Jacksonville, Ill., will, it is said, sail for Europe early in July, in accordance with the plan outlined in an editorial article printed in the last number of this journal.

QUININE, in 1880, sold for three dollars per ounce. The price is now fifty-two cents. The reduction is due to artificial cultivation of the Cinchona tree, and to the removal of tariff duties.

DR. ZAWODSKI mentions in a Polish Journal a case where the extraction of a lower wisdom tooth was followed by death from pyæmia on the nineteenth day.

